# Supplementary material for: High performance Legionella pneumophila source attribution using genomics-based machine learning classification
Source: Appl Environ Microbiol. 2024 Jan 30;90(3):e01292-23. doi: 10.1128/aem.01292-23 (PMC10952463; doi:10.1128/aem.01292-23)
Supplement: Supplementary Figure S1 — Evolutionary rooted maximum likelihood tree of 534 L. pneumophila genomes with tips individually labeled with the isolate ID and outbreak group. [file aem.01292-23-s0001.docx]

**Supplementary Figure 1.** Evolutionary rooted Maximum Likelihood tree of 534 *L. pneumophila* genomes with tips individually labelled with the isolate ID and outbreak group.
